# Buffalo Medical Association

**Published:** 1880-02

**Authors:** 


### SOCIETY REPORTS.

---

### BUFFALO MEDICAL ASSOCIATION.

*Stated Meeting, January 6, 1879.*

THE PRESIDENT, DR. LUCIEN HOWE, IN THE CHAIR.

MEMBERS present, Drs. Fowler, Wyckoff, Cronyn, Gould, Mynter, Moody, Gay, Trowbridge, Keene, Bartlett and Hartwig.

Dr. C. C. F. Gay read an interesting paper on "Refracture for the Correction of Deformity." The reasons for the procedure were given in detail, the method described, and cases cited in which its employment had proved beneficial.

In the discussion which followed, Dr. Mynter spoke favorably of refracture, though he differed with Dr. Gay as to the universality of its application.

Dr. Cronyn thought it would be much better if injuries were so treated in the first place, as to result in slight deformity. He feared that serious harm might often result to contiguous vessels and nerves by the act of refracture, and thought that if any operative interference was to be employed, it should be an osteotomy with Lister's antiseptic precautions.

Dr. Bartlett objected to refracture, because in the majority of cases it was necessary to keep the patient in bed afterwards, and this prolonged confinement, he thought, was sometimes permanently detrimental.

Dr. Hartwig considered osteotomy preferable to refracture, especially if the antiseptic method was used.

Dr. Gay, in reply to these objections said, that according to his experience refracture was not attended by danger to the limb, nor by pain and injury to the health of the patient; indeed, so little contusion was produced, and it was followed by such slight pain or inflammatory symptoms of any kind, that for this reason especially, did he prefer it to any other method.

Among the prevailing diseases, scarlatina and rubeola were reported as particularly frequent, and cases were mentioned by Drs. Cronyn and Bartlett, to show that the two diseases could exist simultaneously in the same patient.

